# Non-invasive Molecular Detection of Minimal Residual Disease in Papillary Thyroid Cancer Patients

**DOI:** 10.3389/fonc.2019.01510

**Published:** 2020-01-10

**Authors:** Hannah Almubarak, Ebtesam Qassem, Lamyaa Alghofaili, Ali S. Alzahrani, Bedri Karakas

**Affiliations:** ^1^Transitional Cancer Research Section, Department of Molecular Oncology, King Faisal Specialist Hospital and Research Center, Riyadh, Saudi Arabia; ^2^Alfaisal University Medical School, Riyadh, Saudi Arabia; ^3^Molecular Endocrinology Research Section, Department of Molecular Oncology, King Faisal Specialist Hospital and Research Center, Riyadh, Saudi Arabia

**Keywords:** thyroid cancer, papillary thyroid cancer, liquid biopsy, minimal residual disease, BEAMing, digital PCR (dPCR)

## Abstract

**Background:** Papillary thyroid cancer (PTC) is the most common type of thyroid malignancy. Serum thyroglobulin (Tg) levels are used to monitor PTC treatment response and recurrences however, in about 25% of the cases the sensitivity of this method is compromised due to either the presence of neutralizing anti-Tg antibodies (TgAb) or the absence of Tg in less differentiated tumors. Up to 80% of PTC tumors harbor the c.1799T>A hotspot mutation in the *BRAF* gene (BRAF^V600E^). Here, we assessed the potential use of plasma cell-free BRAF^V600E^ mutant tumor DNA (ctDNA) levels in determining the minimal residual tumor status of PTC patients.

**Methods:** Patients were classified as either having persistent disease (PD) or no evidence of disease (NED) based on clinicopathological assessments. Tumor BRAF^V600E^ status was determined by both direct sequencing and digital PCR. Plasma total cell-free BRAF^V600^ wild type DNA (cfDNA) and ctDNA fractions circulating in the plasma of PTC patients were determined by an emulsion based-digital PCR and total ctDNA was quantified by 3D digital PCR. The total ctDNA levels (copies/ml) were then compared to patients' clinicopathological features.

**Results:** About 74% (28/38) of tumors harbored the BRAF^V600E^ mutation. Percent plasma ctDNA fractions for PD patients with BRAF^V600E^ tumors ranged from 0 to 2.07%, whereas absolute plasma ctDNA copies ranged from 0 to 62 copies. The ctDNA levels accurately detected tumor burden of PTC patients whose tumors harbored BRAF^V600E^; median plasma ctDNA copy numbers were significantly higher (Wilcoxon test, *p* = 0.03) in patients with metastasis (MET) (20 copies/ml) compared to patients with non-metastatic (non-MET) tumors (1 copy/ml). The plasma ctDNA levels (copies/ml) accurately determined the disease status of PTC patients with sensitivity of 86% and specificity of 90% as compared to 78% sensitivity and 65% specificity determined by serum Tg levels (ng/ml) with areas under the curves (AUC) of 0.88 and 0.71, respectively. Intriguingly, plasma total cfDNA levels were significantly higher in patients with no evidence of residual disease (NED) compared to persistent disease (PD) patients.

**Conclusions:** Our study supports the clinical applicability of plasma ctDNA as biomarker to determine the residual tumor status and tumor burden of PTC patients.

## Introduction

The incidence of papillary thyroid cancer (PTC) has dramatically increased in the United States and most other developed countries in the last three decades (1, 2). In addition to early and more frequent detection of PTC due to widespread use of neck ultrasonography, this increase is also attributed to good prognosis and low mortality rates, which translates to over a 90% survival rate. However, about one third of PTC cases recur even after several years of remission thus emphasizing the need for more frequent and effective follow-ups.

The current management of PTCs consists of thyroidectomy with or without radioactive iodine ablation. After the initial treatment, patients need a long-term follow-up for early detection of possible recurrences. The routine follow-up methods mainly consist of ultrasonography and serum thyroglobulin (Tg) measurements (3). Since the initial report in 1975, serum Tg level has become the gold standard tumor marker for detecting persistent/recurrent PTCs after initial therapies. However, in about 25% of the cases, the presence of elevated anti-Tg antibodies (TgAb) interferes with most Tg assays making this technique unreliable for clinical assessment of PTCs (4, 5). In addition to harmful radiation exposure, radiological techniques also suffer from low sensitivity for the early detection of low tumor masses (6). Therefore, there is a clear need for additional more sensitive and safe molecular assays to efficiently monitor PTC patients.

Even though blood plasma cell-free DNA was first reported in the late 1940s, its use for diagnosis has been only recently realized (7–10). With the advent of more sensitive molecular technologies, detecting a minute amount of cell-free tumor DNA (ctDNA) in the plasma of cancer patients opened new avenues for early diagnosis and treatment follow-ups (11).

Acquired functional DNA mutations in tumor suppressors and oncogenes are known to be the major causes for most tumorigenesis (12). These mutations can also serve as tumor biomarkers for cancer diagnosis, prognosis, and monitoring relapses. The c.1799T>A hotspot mutation in the *BRAF* gene (BRAF^V600E^) is highly frequent in PTCs (10, 13, 14).

In our study, to assess the potential use of plasma ctDNA levels in determining minimal residual tumor status of papillary thyroid cancer (PTC) patients, we quantified plasma ctDNA molecules by an emulsion-based PCR mutation detection assay (BEAMing) and 3D digital PCR in a cohort of 38 PTC patients targeting the BRAF^V600E^ hotspot mutation site. Our findings were then compared to the patients' clinicopathological features including serum thyroglobulin (Tg) levels at the time of blood sampling. We were able to detect residual PTC tumors by measuring the ctDNA in the plasma of PTC patients undergoing post thyroidectomy treatment and follow-up.

## Results

In this proof-of-principle study, we first determined the BRAF^V600E^ hotspot mutation status in tumors of 38 PTC patients both by direct sequencing and digital PCR. We detected the BRAF^V600E^ mutation in 28 out of 38 PTC tumor samples (73.6%). We then determined the percent plasma cell-free BRAF^V600E^ mutant tumor DNA (ctDNA) fractions in the background of cell-free BRAF^V600^ wild type DNA (cfDNA) by the BEAMing assay and finally estimated the total plasma ctDNA copies from percent ctDNA for all the 38 patients. The PTC patients were classified as having persistent disease (PD) and no evidence of disease (NED) based on radiological and other clinicopathological assessments combined if necessary; of the 38 PTC patients, 18 patients had PD and 20 had NED. Seventy-eight percent of patients with PD harbored BRAF^V600E^ hotspot mutation in their tumors.

### Digital PCR Detected Tumor BRAF^V600E^ Mutation With a Higher Sensitivity

To use the hotspot mutation as a molecular marker, we analyzed the tumors from all 38 PTC patients for their BRAF^V600E^ mutation status both by Sanger sequencing and 3D digital PCR (QuantStudio 3D, Applied Biosystems) techniques. The QuantStudio 3D detected the BRAF^V600E^ mutation in 28 out of the 38 tumors (73.6%) while Sanger sequencing detected BRAF^V600E^ in only 20 tumors (52.6%).

### Percent Plasma ctDNA Predicts Disease Status

**Figure 1:**
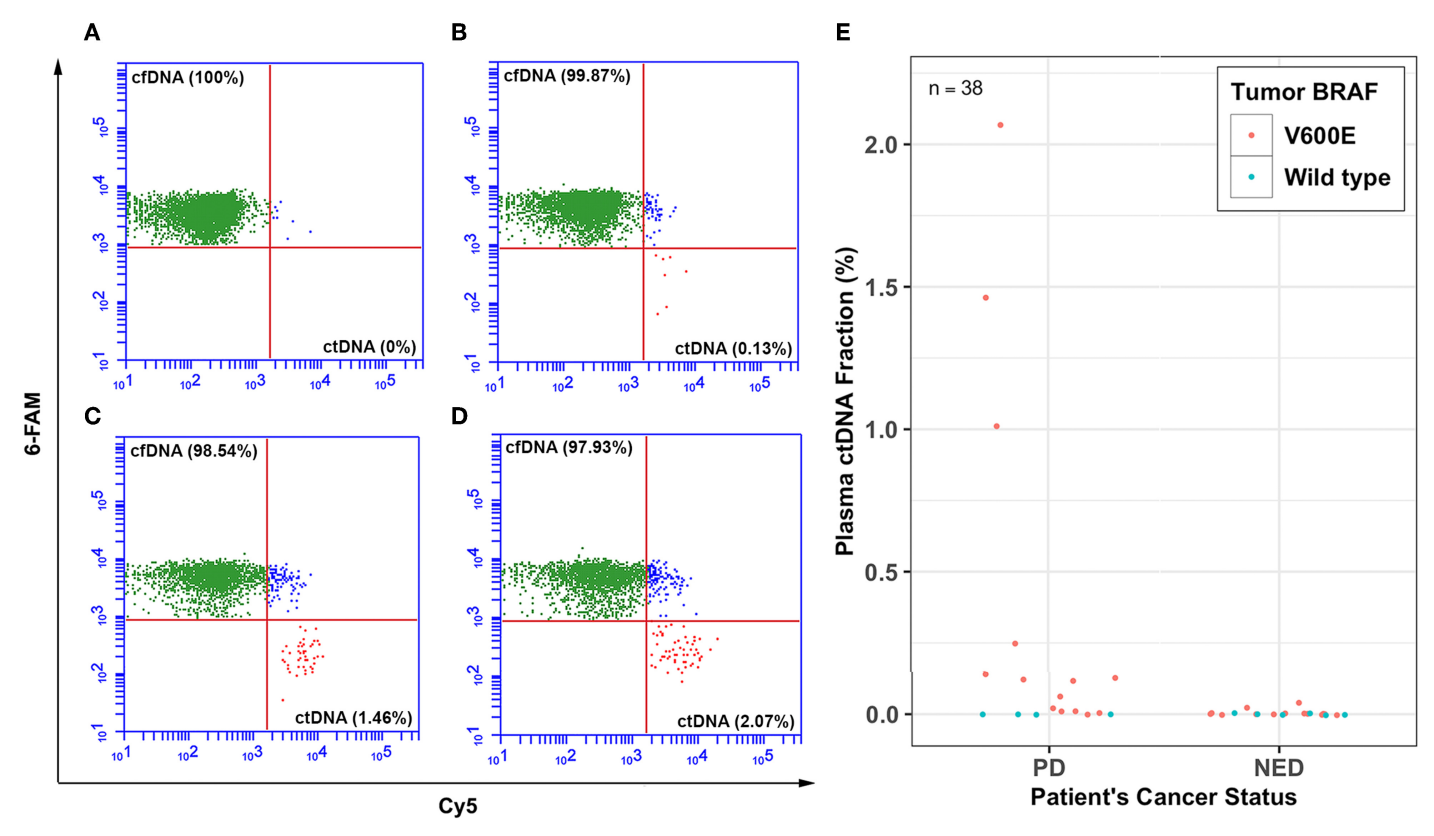
Percent plasma ctDNA predicts disease status. Representative FACS plots for selected patients showing the percent (%) plasma cell-free BRAFV600 wild type (cfDNA) and mutant DNA (ctDNA) levels for thyroid cancer patients whose tumors harbored BRAF^V600E^ hotspot mutation with various levels of disease; **(A)** plasma ctDNA (0%) level for a patient with no evidence of disease (NED), **(B)** plasma ctDNA level (0.13%) for a patient with PD and no evidence of distant metastasis, **(C)** plasma ctDNA level (1.46%) for a patient with lung metastasis, and **(D)** plasma ctDNA level (2.07%) for another patient who also had lung metastasis. **(E)** Dot plot showing percent ctDNA levels for all 38 patients with either PD or NED.

The percent plasma ctDNA and cfDNA circulating in the plasma of patients were determined by the BEAMing technique and results were quantified by FACS analyses (Figures 1A–D). Figure 1 shows the FACS analysis of four representative patients with various cancer status; a patient with NED was found to have no detectable plasma ctDNA (Figure 1A), a patient with PD and no known metastasis had 0.13% plasma ctDNA (Figure 1B), PD patients with lung metastasis had 1.46 and 2.07% plasma ctDNA (Figures 1C,D), respectively. The percent plasma ctDNA level was found to be as high as 2.07% of the total plasma circulating cfDNA for patients with PD and distant metastasis while there was no detectable ctDNA in the plasma of patients who were classified as NED except for two patients. The overall plasma ctDNA fractions in patients with PD and those with NED whose tumors were positive for the BRAF^V600E^ hotspot mutation ranged from 0 to 2.07% and 0 to 0.04%, respectively (Figure 1E).

### Plasma ctDNA Levels Detected Patient's Disease Status and Tumor Burden

**Figure 2:**
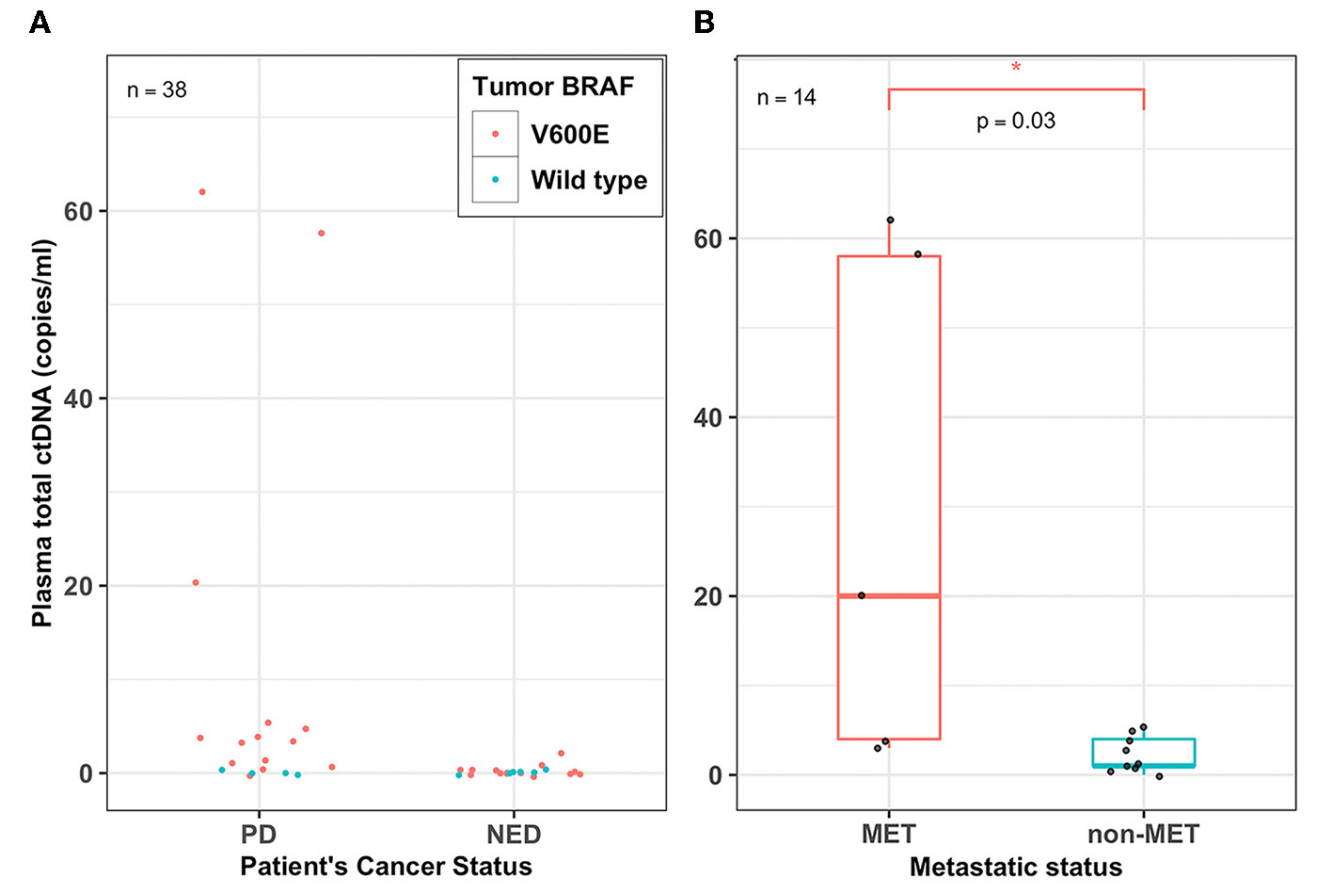
Plasma ctDNA levels detected patient's disease status and tumor burden. **(A)** Dot plot showing the overall plasma cell-free tumor DNA (ctDNA) levels (copies/ml) for all the 38 PTC patients who had either persistent disease (PD) or no evidence of disease (NED). Each filled circle represents a value from a patient; red-filled circles represent patients whose tumors harbor the BRAF^V600E^ mutation and green-filled circles represent patients whose tumors harbored wild type BRAF, **(B)** A box plot showing the range and median percent plasma ctDNA levels (copies/ml) of the PD patients whose tumors harbored the BRAF^V600E^ mutation and were either metastatic (MET) or non-metastatic (non-MET). Plasma total ctDNA levels were significantly higher in MET patients (median, 20 copies/ml) compared to that of non-MET patients (median, 1 copy/ml) (Wilcoxon test, *p* = 0.03; *n* = 14). *p < 0.05.

From the ctDNA fractions, we estimated the absolute ctDNA copies for patients whose tumors harbored the BRAF^V600E^ hotspot mutation. The overall ctDNA copies for patients with either PD or NED ranged from 0 to 62 copies or 0 to 2 copies per milliliter of plasma, respectively (Figure 2A). Additionally, plasma ctDNA levels accurately estimated patients' cancer stage (i.e., metastatic vs. non-metastatic). The ctDNA levels of patients whose tumors were metastatic (MET) were found to be significantly higher (Wilcoxon test, *p* = 0.03) than those with non-metastatic tumors (non-MET) with a median of 20 copies and 1 copy per milliliter for MET and non-MET patients, respectively (Figure 2B).

### Plasma cfDNA Levels Are Higher in NED Patients

**Figure 3:**
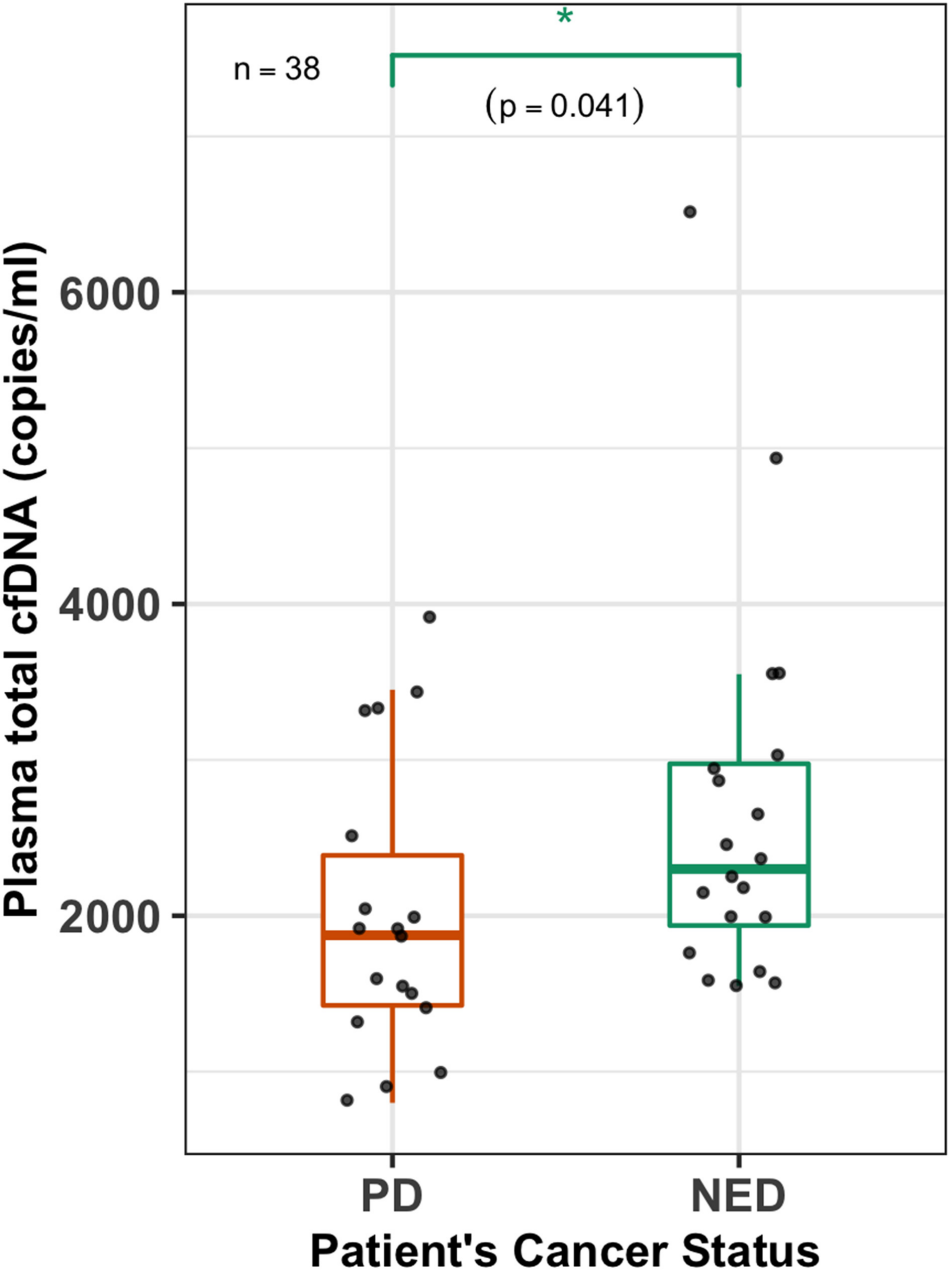
Plasma cfDNA levels are higher in NED patients. A box-plot overlaid by a dot-plot showing the range and median values of plasma total cell-free DNA (cfDNA) (copies/ml) levels for all the patients (*n* = 38) that were estimated by the 3D digital PCR. Plasma total cfDNA levels are significantly higher (Wilcoxon test, *p* = 0.041, *n* = 38) in patients with no evidence of disease (NED) compared to those who had persistent disease (PD). *p < 0.05.

The total plasma cfDNA copies (copies/ml) (both mutant and wild type) were also quantified for all 38 PTC patients. The cfDNA levels for the *BRAF* gene of PTC patients ranged from 800 to 6,500 copies (copies/ml) with a mean and median of 2,363 and 2,025 copies, respectively. Patients with PD were found to have a lower median plasma cfDNA (copies/ml) compared to that of patients with NED (1,875 vs. 2,300) (Wilcoxon test, *p* = 0.041; Figure 3).

### Serum Levels of PD and NED Patients

We measured the serum Tg level (ng/ml) of each patient and found that the overall blood serum Tg levels for our patient population ranged from <0.1 to 86 ng/ml. The serum Tg levels for PD and NED patients ranged from <0.1 to 86 and <0.1 to 9 ng/ml, respectively (Figure S1, Table S1).

### Moderate Association Between Patients' Serum Tg and Plasma ctDNA Levels

**Figure 4:**
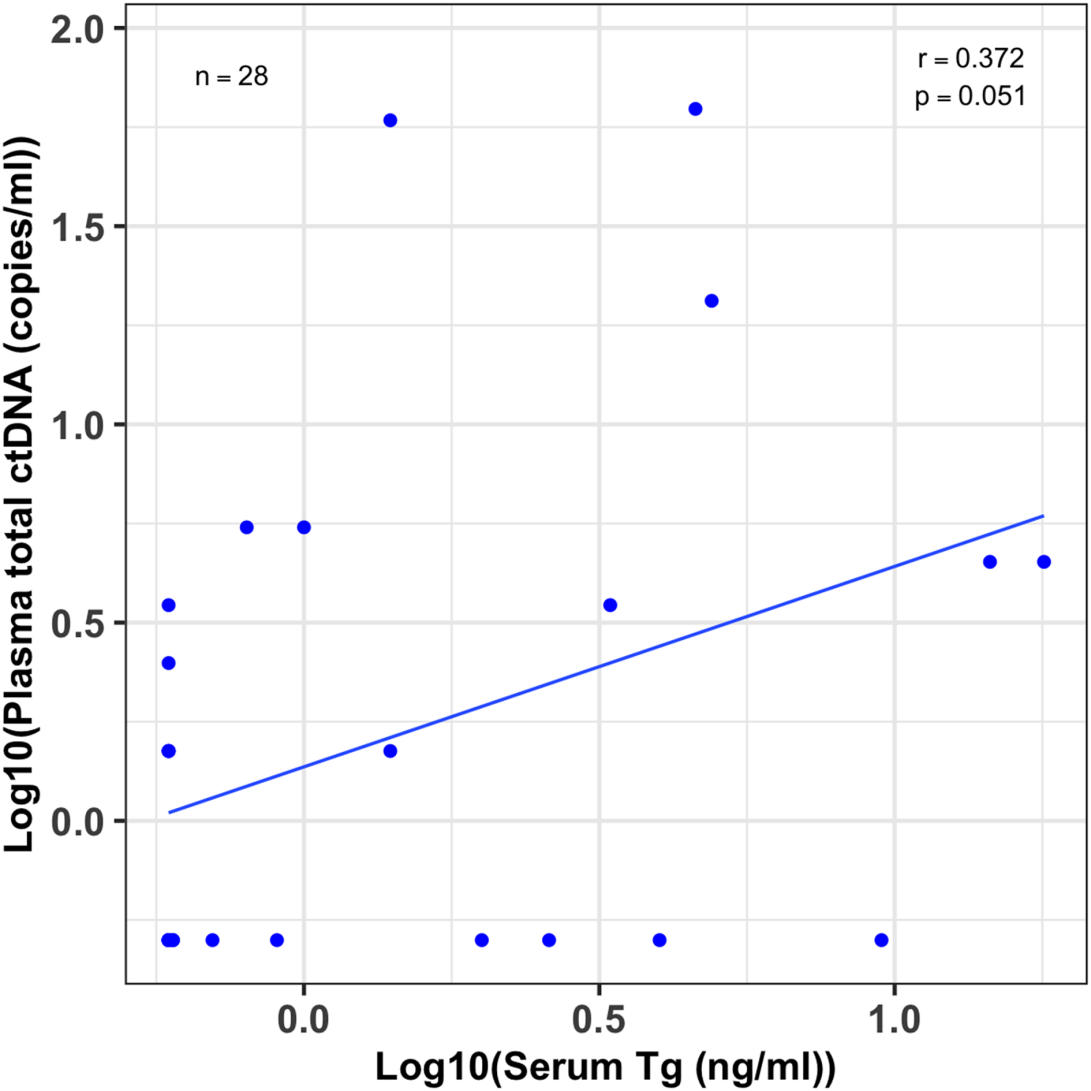
Moderate association between patients' serum Tg and plasma ctDNA levels. A scatter plot showing the correlation between serum thyroglobulin (Tg) levels and their plasma total cell-free tumor DNA (ctDNA) levels for patients whose tumors harbored the BRAF^V600E^ mutation (*n* = 28). Serum Tg (ng/ml) and plasma ctDNA (copies/ml) data was log10 transformed before the correlation analysis. The correlation coefficient (r) and *p*-value were estimated by Pearson's correlation test. Even though the sample size was small there is a modest correlation between patients' serum Tg and plasma ctDNA levels (*r* = 0.37; 95% CI, 0–0.65, *p* = 0.051).

To evaluate the degree of association between plasma ctDNA and serum Tg levels of the same patients, we first log transformed both the ctDNA and Tg data for normalization, and then estimated the Pearson's correlation coefficient (r). We compared the serum Tg levels and plasma total ctDNA values for the 28 patients whose tumors harbored the BRAF^V600E^ hotspot mutation. Even with a relatively small sample size, we found a moderate correlation between Tg levels and ctDNA copies with a correlation coefficient of *r* = 0.37 and a *p*-value of 0.05 (Figure 4).

### Plasma ctDNA Levels Perform Better Than Serum Tg Levels in Diagnosis of PTC Patients

**Figure 5:**
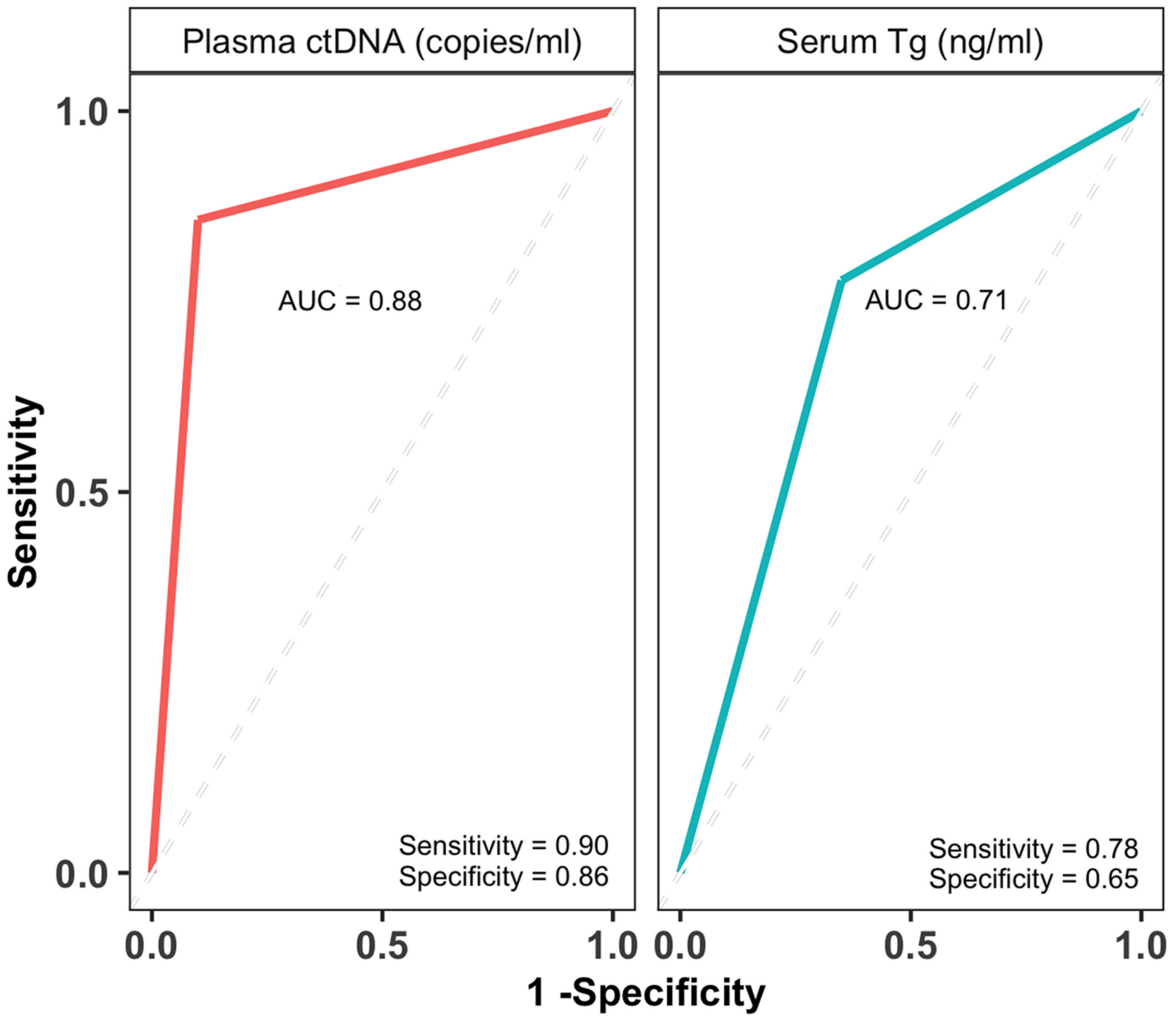
Plasma ctDNA levels perform better than and serum Tg levels in diagnosing PTC patients. The receiver operating characteristic (ROC) curves and area under the curve (AUC) of plasma ctDNA (copies/ml) and serum Tg levels (ng/ml). The AUCs are indicated on each curve along with sensitivity and specificity values.

The BEAMing technique accurately determined the disease status of PTC patients with 86% sensitivity (95% CI, 0.57, 0.98) and 90% specificity (95% CI, 0.68, 0.99) whereas the sensitivity and specificity of the serum Tg assay was 78% (95% CI, 52, 94) and 65% (95% CI, 0.41, 0.85), respectively (Table S2). The receiver operating characteristics (ROC) curves were generated by plotting the number of true positive rates (Sensitivity) against false positive rates (1-Specificity) for both ctDNA and Tg predictions. The area under the curve (AUC) for ctDNA and Tg levels were estimated from ROC curves. The AUC for ctDNA levels (0.88) was higher than that of Tg levels (0.71) (Figure 5), which indicates that ctDNA based PTC diagnosis performs better than that of Tg levels.

### Patients' Tumor BRAF^V600E^ Hotspot Mutation Status Did Not Affect Plasma cfDNA Levels

To see if the patients' cfDNA levels are influenced by the tumor BRAF mutation status, we compared the cfDNA levels of patients whose tumors harbored the BRAF^V600E^ mutation and those with wild type tumors. There was no significant difference (Wilcoxon test, *p* = 0.67) between the median cfDNA levels (copies/ml) for patients whose tumors harbored the BRAF^V600E^ mutation and patients whose tumors had wild type BRAF^V600^ (2,000 vs. 2,350) (Figure S2).

### Patients' Disease Status and Tumor BRAF^V600E^ Mutation Status

We also compared the frequencies of PTC patients' disease status to their tumor BRAF hotspot mutation status. Seventy-eight percent (14/18) of patients with PD had BRAF^V600E^ positive tumors while 70% (14/20) of NED patients harbored the BRAF^V600E^ mutation. We observed a higher number of PD patients who had tumor BRAF^V600E^ mutation compared to those of NED, however this difference was not statistically significant (Fisher's Exact test, *p* = 0.72) (Figure S3).

## Discussion

Here, we determined the disease status of PTC patients by measuring their plasma ctDNA levels and compared it to their clinicopathological features including serum Tg levels. Our main findings are summarized in the following points; first, the BEAMing assay was able to accurately detect the disease status of PTC patients with over 86% sensitivity and 90% specificity; second, plasma ctDNA levels predicted the tumor burden; third, we found that the overall total cfDNA levels of NED patients were higher than those of PDs. Lastly, there was a moderate correlation between serum Tg and plasma ctDNA levels.

We found a relatively higher sensitivity and specificity of plasma ctDNA detection using BEAMing compared to that of previous studies (13). This could be due to the power of the BEAMing technique over the other commercially available digital PCR assays. The sensitivity and specificity of measuring the plasma ctDNA fractions and the absolute ctDNA copies was also higher than that was found by measuring serum Tg levels for the same patients. Interestingly, total cfDNA levels were significantly higher in NED patients. It seems that the age and gender of the patients does not explain this difference. The male subjects overall have higher cfDNA levels than female subjects as previously reported (10). We have similar numbers of male subjects in PD and NED patient categories however cfDNA levels of male subjects in NED patients is also higher.

We found a modest correlation between the plasma ctDNA copies and serum Tg levels. While liquid biopsies based on ctDNA quantification depend on the presence of functional tumor mutations, Tg antigen based assays have their own shortcomings such as low detection levels (0.1 ng/ml) and the presence of neutralizing anti-TgAb in about a quarter of the PTC cases (5). We believe the combined plasma ctDNA quantities and serum Tg levels will result in a much higher sensitivity and specificity approach when detecting minimal residual tumors. Similar to multi-drug treatments, multi-analyte based diagnosis is likely to have a higher predictive value for cancer patients (14).

Currently, there are many ongoing clinical trials assessing the plasma ctDNA levels for the clinical management of various cancer types (15). Thus, we believe that ctDNA quantification based liquid biopsies are soon to become a widely available method in early cancer recurrence detection and response to treatment. Recently, a liquid biopsy based medical test that detects several clinically significant EGFR somatic tumor mutations in the plasma of non-small cell lung cancer (NSCLC) patients has been approved by the FDA (16).

The plasma ctDNA levels alone however are not sufficient to make clinical decisions regardless of the sensitivity of the technique used. In a recent study plasma ctDNA levels along with a set of plasma proteins were analyzed for early detection in over 1,000 patients of eight cancer types (14). The utilization of ctDNA in clinical practice depends on the sensitivity and specificity of the technique used, the type of cancer, and whether the tumor is localized or metastasized to distant sites (17).

The prerequisite of using plasma ctDNA as a liquid biopsy is based on the availability of at least one causative gain or loss-of-function somatic mutation in the coding region of a gene that drives early tumorigenesis. In our study population, about 74% of PTC patients harbored the BRAF^V600E^ hotspot mutation in their tumors. However, not all cancer types have such high frequencies of somatic hotspot mutations. Therefore, for those cancers without high frequency hotspot mutations, next generation sequencing could be adopted as a non-invasive detection method (18).

In conclusion, our study suggests that it could be possible to use plasma ctDNA to determine the presence of minimal residual tumors in PTC patients with a higher sensitivity and specificity than the assays currently used in the clinical management of thyroid cancers however this needs to be further validated in clinical studies with larger sample size. We also believe both plasma ctDNA and serum Tg levels could be used in combination to achieve higher sensitivities and specificities in determining the PTC status.

## Materials and Methods

### Patient Population

This study consists of 38 PTC patients who were undergoing treatment at King Faisal Specialist Hospital and Research Center (KFSH&RC). Of the 38 PTC patients, there were 29 females and nine males with their ages ranging from 21 to 74 years with a median of 42.5 years of age. Of the 38 PTC patients, the tumors of 28 patients were positive for the BRAF^V600E^ mutation and 10 had wild type BRAF^V600^ tumors. Based on clinicopathological evaluations 20 patients were deemed as having no evidence of disease and 18 had persistent disease. Suspicious sonographic appearances for persistent cases were further confirmed with fine needle aspiration (FNA) biopsies and/or Tg measurements. Formalin-fixed and paraffin embedded (FFPE) tumor tissues and blood samples from the 38 PTC patients were collected (Table S1). Fresh blood (2 ml) samples were drawn into BD Vacutainer EDTA tubes (Cat# 366643), immediately centrifuged at 3,000 rpm for 10 min at +4°C, and the plasma fractions were transferred to a clean tube and re-centrifuged at top speed for another 10 min and finally the plasma was carefully transferred to a clean tube to avoid any possible cell pellet and stored at −80°C.

### Tumor Genomic DNA (gDNA) and Plasma Total Cell-Free (cfDNA) Isolation

Genomic DNA was extracted from FFPE tumor sections using QIAamp DNA FFPE Tissue Kit (Qiagen, Cat#56404) by following the manufacturer's protocol. About 2–3 sections were treated with 1 ml xylene to remove the paraffin, tumor cell pellet was then lysed and DNA was eluted in 50 μl elution buffer and stored at −20°C for further analysis. Total cfDNA was isolated from plasma samples using QIAamp Circulating Nucleic Acid Kit (Qiagen, Cat#55114) following the modified manufacturer's protocol previously described except that the cfDNA was eluted in 50 μl volume (19).

### Identification of Patient's Tumor BRAF^V600E^ Mutation Status

#### Sanger Sequencing

The coding region of the *BRAF* gene spanning the BRAF^V600^ site was PCR amplified using target specific primers containing M13 tail sequences (Table S3). Both M13 forward and reverse primers were used for bidirectional sequencing of the amplified PCR products. PCR products were sequenced at the Genomic Core Facility of KFSH&RC.

#### QuantStudio 3D Digital PCR Verification

In addition to Sanger sequencing, patient tumor status was identified and also verified for the BRAF^V600E^ hotspot mutation by a 3D digital PCR assay. About 10 ng DNA extracted from each FFPE tumor tissue was used in 14.5 μl of 3D digital PCR reaction mixture. The detailed protocol for the 3D digital PCR technique is described in the following section.

### Determining the Plasma ctDNA Fraction

Plasma ctDNA fractions were determined by two subsequent PCR reactions: First, the gene region spanning BRAF^V600^ site was PCR amplified using high fidelity *Phusion polymerase* enzyme and specific primers generating about a 130 bp fragment size. Then, this PCR product was used as a template along with specific primers to amplify single copy templates on individual beads during the BEAMing reaction. The overall protocol for BEAMing was followed as previously described with the exception of few modifications (19, 20).

Briefly, about 20 μl of the 35 μl DNA extracted from one-milliliter plasma was PCR amplified in four separate PCR reactions, pooled, and cleaned using PCR clean-up beads (cleanPCR Cat#: CPCR-0050) and re-suspended in 40 μl sterile water. The copy numbers of the purified initial PCR product were estimated by the 3D digital PCR assay. About 60–70 million DNA copies (10–20 μl) from the initial PCR product were added in a total of 160 μl BEAMing reaction volume. All the primers and probes used in both initial PCR and BEAMing steps are listed in Table S3. Percent plasma mutant ctDNA fractions for patients were determined by FACS analysis (Figure 1) and the total ctDNA copy numbers were estimated from the BEAMing mutant fraction and plasma total cfDNA copy numbers estimated by 3D digital PCR for each sample.

### Determining Plasma Total cfDNA and ctDNA Copies

Plasma total cfDNA copy number for each patient was quantified by the QuantStudio 3D digital PCR system using primers (Table S3) spanning the BRAF^V600^ region. Digital PCR reaction mix was prepared in a total volume of 17.4 μl for a sample as follows: 8.7 μl 2X digital PCR master mix, 0.85 μl BRAF-V600E and BRAF-wild type TaqMan probe mix (Cat # 4383547/AH6R5PH BRAF_476), 5 μl of plasma cfDNA (2–2.5 ng), 0.4 μl of 100 μM custom primer mix, 2.45 μl nuclease free water. About 14.5 μl of the mix was loaded onto the chip using Chip Kit V2 and QuantStudio 3D digital PCR chip loader. The chip was then placed on ProFlex 2x Flat PCR system and reaction amplification was performed using the following cycle settings: (96°C for 10 min) × 1 cycle; (98°C for 30″, 60°C for 2′) × 39 cycles; and (60°C for 2′) × 1 cycle. Following the amplification process, chips were analyzed using QuantStudio Digital PCR Chip Reader (ThermoFisher, Cat# 4489084) and the data was uploaded into the QuantStudio 3D AnalysisSuite Cloud Software for analysis. Plasma absolute total cfDNA copy numbers (mutant and wild type) were determined as copy number per milliliter of plasma for each sample. Absolute ctDNA copies were estimated from percent ctDNA values and converted to total copies per milliliter of plasma.

### Serum Thyroglobulin (Tg) Measurements

Serum Tg levels (ng/ml) and TgAb (IU/ml) for all the samples were measured using electro-chemiluminescence immunoassay kit (Elecsys Tg II, Roche) in the KFSH&RC Pathology Laboratory following manufacturer's procedures.

### Statistical Analysis

All statistical analysis and figure preparations were done by the publicly available R Studio software (R version 3.5.0) and ggplot2 package. The Tg and ctDNA data was log transformed for association analysis. Wilcoxon rank test was used to compare the plasma total cfDNA levels. The Fisher's exact test was also used to determine any possible association between the frequency of patients' tumor *BRAF* mutation status and their disease status. The ROC curves were drawn and AUCs for ctDNA and Tg level predictions were estimated using plotROC along with ggplot2 R library packages.

## Data Availability Statement

All datasets generated for this study are included in the article/Supplementary Material.

## Ethics Statement

The studies involving human participants were reviewed and approved by The Research Ethics Committee (REC) of King Faisal Specialist Hospital and Research Center. The patients/participants provided their written informed consent to participate in this study.

## Author Contributions

AA and BK: study conception and design and overall supervision. AA and LA: patient recruitment, sample, and clinical data collection. AA and EQ: methodology (tumor DNA extraction and Sanger sequencing). HA and BK: methodology (plasma DNA extraction, BEAMing, 3D digital PCR). BK, AA, and HA: data analysis and interpretation and writing and editing the manuscript.

### Conflict of Interest

The authors declare that the research was conducted in the absence of any commercial or financial relationships that could be construed as a potential conflict of interest.
