# Supplementary material for: Non-invasive Molecular Detection of Minimal Residual Disease in Papillary Thyroid Cancer Patients
Source: Front Oncol. 2020 Jan 10;9:1510. doi: 10.3389/fonc.2019.01510 (PMC6966766; doi:10.3389/fonc.2019.01510)
Supplement: Supplementary file 1 [file Data_Sheet_1.pdf]

Supplementary Material

Noninvasive molecular detection of minimal residual disease in papillary thyroid cancer patients

Hannah Almubarak[1], Ebtesam Qassem[2], Lamyaa Alghofaili[3], Ali S. Alzahrani[2, 4], Bedri Karakas[1]

[1]Transitional Cancer Research Section, Department of Molecular Oncology, King Faisal Specialist Hospital and Research Center, Riyadh, 11211, Saudi Arabia

[2]Alfaisal University Medical School, Riyadh, 1153, Saudi Arabia

[3]Molecular Endocrinology Research Section, Department of Molecular Oncology, King Faisal Specialist Hospital and Research Center, Riyadh, 11211, Saudi Arabia

[4]Molecular Endocrinology Research Section, Department of Molecular Oncology, King Faisal Specialist Hospital and Research Center, Riyadh, 11211, Saudi Arabia

Keywords: thyroid cancer, PTC, liquid biopsy, minimal residual disease, BEAMing, digital PCR

Correspondences:

1. Bedri Karakas, PhD
   E-mail: bedrik@yahoo.com

2. Ali S. Alzahrani, MD
   E-mail: aliz@kfshrc.edu.sa

Figure S1.

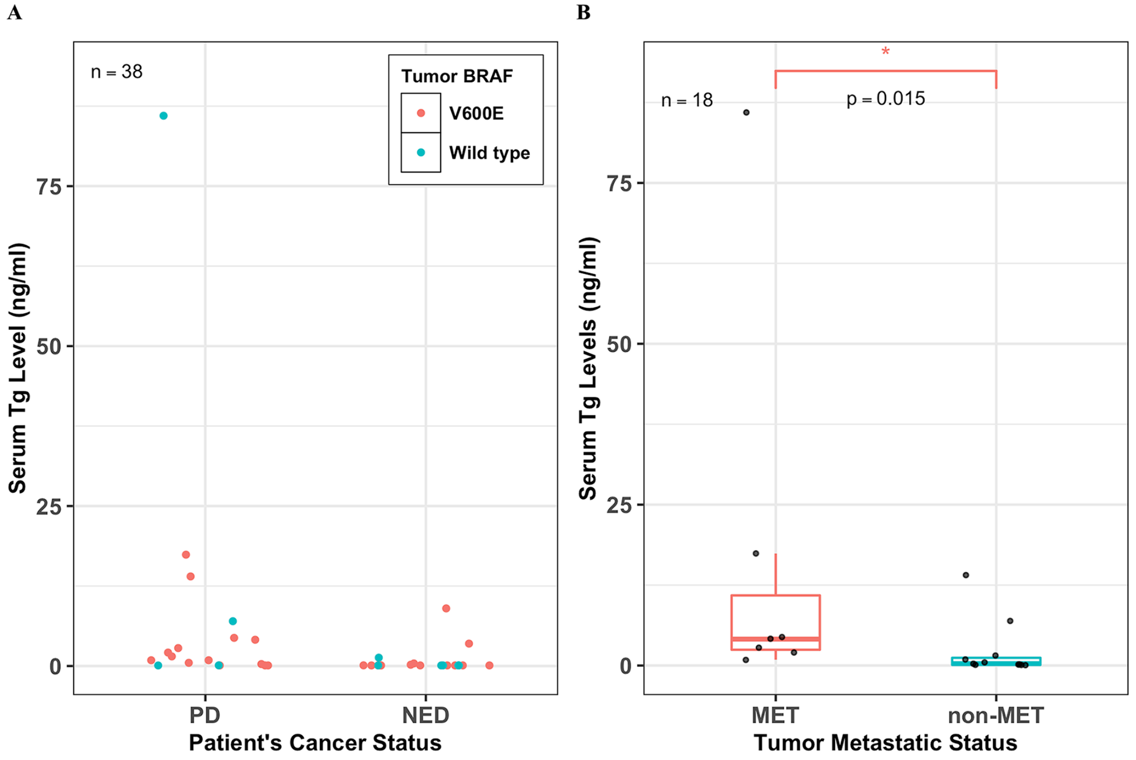

Figure S1. Serum Tg levels and disease status/tumor burden. (A) Dot plot of serum thyroglobulin (Tg) levels (ng/ml) for all the patients (n=38) who were either classified as having persistent disease (PD) or no evidence of disease (NED) based on their clinicopathological features and radiological assessments. **(B)** Box plot showing the range and median serum Tg levels (ng/ml) for patients whose tumors were either metastatic (MET) or non-metastatic (non-MET). The serum Tg levels of patients with MET were significantly higher than that of non-MET patients (Wilcoxon test, p=0.015; n=18).

Table S1. Patients' clinicopathological features.

No	Patient No	Gender	Age	Tumor BRAF status	Blood sample date	Serum Tg (ng/ml)	Serum TgAb (U/ml)	TSH	Disease status	Comments
**1**	**569**	Female	74	V600E	3/30/14	0.9	NA	0.93	PD	Lung metastasis
**2**	**74**	Female	44	V600E	1/12/14	<0.1	133	0.35	PD	
**3**	**168**	Female	61	V600E	1/22/14	4.7	12	0.071	PD	Lung metastasis
**4**	**254**	Male	55	V600E	2/9/14	53.6	<10	>1000	PD	Lung metastasis
**5**	**53**	Female	61	V600E	1/8/14	1.1	2982	53	PD	Lung metastasis
**6**	**313**	Female	35	V600E	2/16/14	1.5	28	0.04	PD	
**7**	**28**	Female	37	V600E	1/5/14	0.5	13	94	PD	
**8**	**70**	Male	21	V600E	1/12/14	4.1	>4000	2	PD	Lung metastasis
**9**	**452**	Male	22	V600E	3/9/14	17.4	27	0.037	PD	FNA positive
**10**	**9**	Female	34	V600E	12/29/13	<0.1	27	0.03	PD	US nodules
**11**	**187**	Male	52	V600E	1/26/14	2.1	20		PD	Lung metastasis
**12**	**378**	Female	48	V600E	2/24/14	<0.1	18	0.026	PD	US nodules
**13**	**255**	Female	52	V600E	2/9/14	0.3	15	332	PD	
**14**	**368**	Female	25	V600E	2/24/14	0.2	14	0.075	PD	US nodules
**15**	**311**	Female	23	Wild type	2/16//14	<0.1	426	3.3	PD	

**16**	**373**	Male	30	Wild type	2/24/14	7	16	4.5	PD	
**17**	**310**	Female	38	Wild type	2/16/14	<0.1	17	0.23	PD	PTC, 1.9 cm
**18**	**126**	Female	42	Wild type	1/19/14	86	<10	68	PD	Lung metastasis
**19**	**75**	Female	47	V600E	1/12/14	<0.1	<10	3.6	NED	
**20**	**496**	Male	67	V600E	3/19/14	0.2	32	0.01	NED	
**21**	**171**	Female	33	V600E	1/26/14	<0.1	<10	1.6	NED	
**22**	**355**	Female	69	V600E	2/23/14	<0.1	14	1.7	NED	
**23**	**154**	Male	52	V600E	1/20/14	0.4	<10	3.3	NED	
**24**	**22**	Female	44	V600E	12/31/13	0.1	17	63	NED	
**25**	**433**	Female	54	V600E	3/3/14	<0.1	172	29	NED	
**26**	**318**	Female	28	V600E	2/16/14	0.1	19	0.048	NED	
**27**	**189**	Male	39	V600E	1/26/14	<0.1	<10	0.11	NED	
**28**	**295**	Female	58	V600E	2/12/14	7	14	0.091	NED	
**29**	**41**	Female	22	V600E	1/6/14	<0.1	115	**0.19**	NED	
**30**	**211**	Female	37	V600E	1/28/14	<0.1	NA	3.5	NED	
**31**	**445**	Male	38	V600E	3/5/14	3.5	29	0.033	NED	
**32**	**283**	Female	57	V600E	2/10/14	9	<10	66	NED	
**33**	**227**	Female	43	Wild type	1/30/14	1.3	15	266	NED	

**34**	**608**	Female	42	Wild type	4/14/14	<0.1	NA	0.01	NED	
**35**	**65**	Female	22	Wild type	1/12/14	<0.1	<10	0.29	NED	
**36**	**366**	Female	65	Wild type	2/23/14	<0.1	12	144	NED	
**37**	**454**	Female	64	Wild type	3/9/14	<0.1	47	44	NED	
**38**	**268**	Female	32	Wild type	2/9/14	<0.1	14	135	NED	

Figure S2.

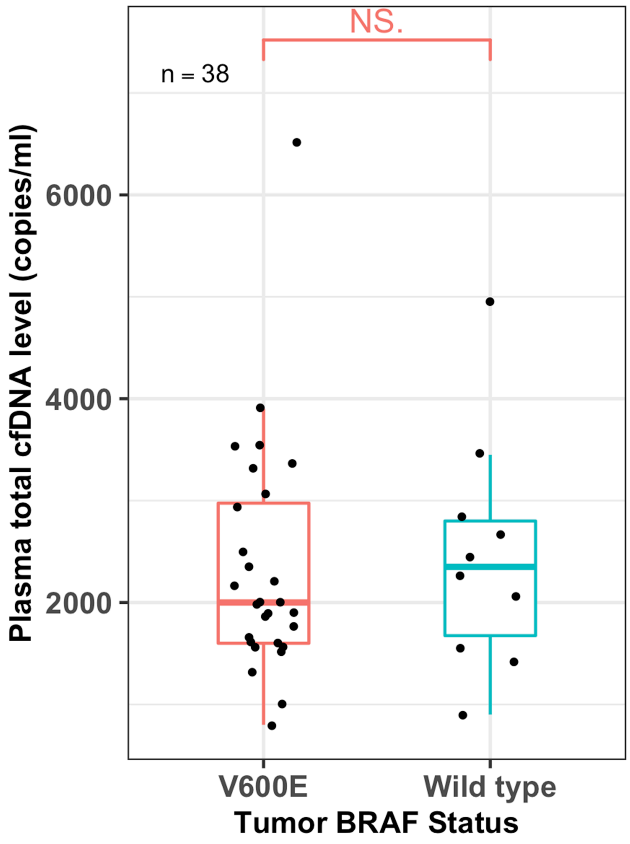

Figure S2. Patients' tumor BRAF^{V600E} hotspot mutation status did not affect plasma cfDNA levels. Plasma total cell-free DNA (cfDNA) of thyroid cancer patients (n=38) whose tumors were either mutant or wild type for BRAF^{V600E} hotspot mutation. There is no difference (Wilcoxon test, $p=0.67$) between the mean plasma cfDNA levels of patients with BRAF^{V600E} positive (n=28) or wild type tumors (n=10).

Figure S3.

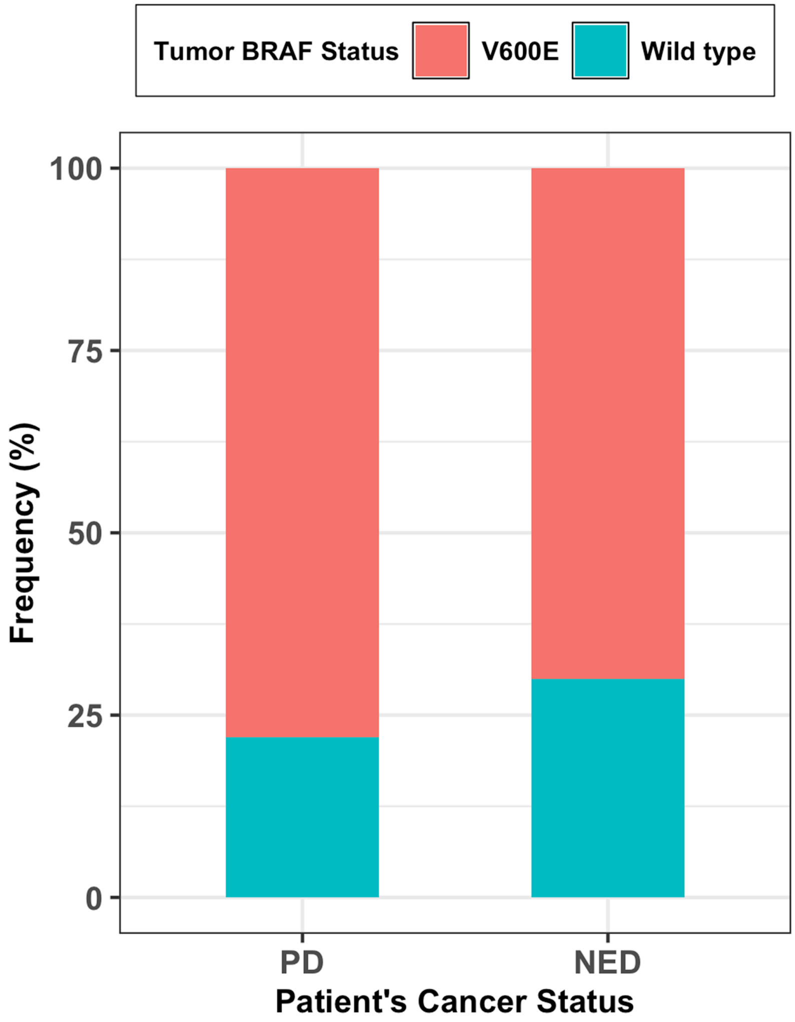

Figure S3. Patients' disease status and tumor BRAF^{V600E} mutation status. The frequency (%) of patients with persistent disease (PD) and patients with no evidence of disease (NED) based on their tumor BRAF^{V600E} hotspot mutation status. Patients with BRAF^{V600E} positive tumors tend to be more resistant (PD) to the therapy but this observation is not statistically significant (Fisher's Exact test, p-value=0.72).

Table S2. The sensitivity and specificity of plasma ctDNA (copies/ml) measured by BEAMing and serum Tg levels (ng/ml) measured by electro-chemiluminescence immunoassay.

**Plasma ctDNA (copies/ml)**			Tumor BRAF	**Cancer status (n)**			
				**PD**	**NED**	%	(95% CI)
**Sensitivity**	**Cancer status by ctDNA levels (n)**[1]	**PD**	V600E	12 (TP)	NA	**86**	**(57, 98)**
		**NED**	V600E	2 (FN)	NA		
	**Total**			**14**	**NA**		
**Specificity**	**Cancer status by ctDNA levels (n)**[1]	**PD**	V600E	NA	2 (FP)	**90**	**(73,99)**
		**NED**	V600E	NA	12 (TN)		
		**NED**	Wild type	NA	6 (TN)		
	**Total**			**NA**	**20**		

**Serum Tg levels (ng/ml)**			**Cancer status (n)**			
			**PD**	**NED**	%	(95% CI)
**Sensitivity**	**Cancer status by Tg levels (n)**[2]	**PD**	14 (TP)	NA	**78**	**(52, 94)**
		**NED**	4 (FN)	NA		
	**Total**		18	NA		
**Specificity**	**Cancer status by Tg levels (n)**[2]	**PD**	NA	7 (FP)	**65**	**(41, 85)**
		**NED**	NA	13 (TN)		
	**Total**		NA	**20**		

Abbreviations: ctDNA: cell-free tumor DNA, Tg: thyroglobulin, PD: persistent disease, NED: no evidence of disease, TP: true positive, FP: false positive, TN: true negative, FN: false negative, NA, not applicable.

[1]Patients whose total estimated plasma ctDNA is > 0 (copies/ml) are considered positive

[2]Patients whose serum Tg levels is ≥ 0.1 (ng/ml) are considered positive.

Table S3. Primers and probes used in pre-BEAMing amplification and BEAMing assay.

Name	Sequence*	Details
**M13-V600 Seq-F**	TCATAATGCTTGCTCTGATAGGA	forward primer for BRAF PCR amplification/sanger sequencing
**M13-V600 Seq-R**	CGGCCAAAAATTTAATCAGTGGA	reverse primer for BRAF PCR amplification/sanger sequencing
**Tag1**	TCCCGCGAAATTAATACGAC	5'-double biotinylated forward primer
**Tag2**	GCTGGAGCTCTGCAGCTA	reverse primer adaptor sequence
**BRAF-F**	TCCCGCGAAATTAATACGATTTCTTCATGAAGACCTCACAG	BRAF gene specific forward primer used for pre-BEAMing amplification and QauntStudio 3D assay (amplicon size=131 bp)
**BRAF-R**	GCTGGAGCTCTGCAGCTAATAGCCTCAATTCTTACCATCC	BRAF gene specific reverse primer used for pre-BEAMing amplification and QuantStudio 3D assay (amplicon size=131 bp)
**BRAF-WT**	TCAAGGTGCGCTGAGAAGATTTCACTGTAG	BRAF wild type allele specific reverse primer used in BEAMing reaction (amplicon size=60 bp)
**BRAF-MT**	TTCGCCGTCAGCCAGTAGATTTCTCTGTAG	BRAF mutant allele specific reverse primer used in BEAMing reaction (amplicon size=60 bp)
**BRAF-WT-FAM**	TCAAGGTGCGCTGAGA	5' FAM -labeled probe specific to BRAF wild type tail sequence
**BRAF-MT-Cy5**	TTCGCCGTCAGCCAGT	5'-Cy5-labled probe specific to BRAF mutant tail sequence

* Underlined nucleotides are the *BRAF* gene specific sequence.
